# Business Notes

**Published:** 1871-01

**Authors:** 


### BUSINESS NOTES.

WHERE TO GO FOR HOLIDAY GOODS.

AT HALL BROTHERS' NEW STORE you will find the largest stock of fancy articles in the city. They have purchased largely for the holiday trade, and have on exhibition toilet sets, writing desks, music holders, fancy ink stands, stereoscopic views and glasses, chess-men, backgammon boards, fancy cutlery, smoking-cases, shaving-cases, bezeque sets, initial stationery, gift books, toy books, elegantly bound volumes of the different authors of prose and poetry, and in fact, any quantity of articles suitable for presents for ladies, gentlemen, or children. Their store and stock is large enough to afford all an opportunity for buying without having long to wait. Call and inspect their goods.

FINE ART GALLERY.—ROOSA & MOULTON at No. 6 Lake St., have an elegant establishment, an ornament to our city, and one of its principal attractions. The pictures to be found here on exhibition are selected with great care and excellent taste. We have never seen any better chromos than those offered at this gallery, and the prices are precisely the same as in New York City. They have been adding largely to their stock of Chromos, Lithographs, Stereoscopic views, fancy brackets, &c., &c., purposely for the holiday trade. A beautiful picture is always a handsome and suitable reminder to the recipient, of the donor's friendship and good taste. Do not fail to call then, at the Art Gallery, and while there examine the splendid gold and velvet frames which they are making. They are the most beautiful we have ever seen. They also keep a large assortment of black walnut goods, such as brackets, cornices, match safes, paper and music holders, &c., &c. Also mirrors and wax work materials. Take a peep at their windows and observe the handsome display of chromos and oil paintings. They are beautiful to behold. Then step inside and see the great variety of articles that may be purchased for presents, and suited to the taste and purse of almost any one.

COLLINGWOOD & STRANG.—Henry has just returned from New York with his usual large stock of holiday goods. He has invested largely in ladies' watches, chains, and jewelry. Has many handsome articles in bronze, suitable for gentlemen or ladies. His Cameo sets are beautiful and are the prevailing style of jewelry. We also find many new and pretty styles of jet jewelry—but you can call and examine the stock yourself; do so by all means, and select presents for wife or sweetheart.

BIOGRAPAICAL DICTIONARY OF DECEASED AMERICAN PHYSICIANS.—Dr. J. M. Toner, of Washington, D. C., is desirous of obtaining information which will aid him in the publication of a Biographical Dictionary of Deceased American Physicians. The work is already in a state of considerable forwardness, and is designed to contain a biographical sketch of every deceased practitioner of regular medicine, from the earliest settlement of our country to the present time.

Hundreds of physicians have died in different parts of the United States, after devoting a long and useful life to their profession, of whose existence and labors there is no record. This should not be so. The want of a work furnishing an outline of the lives of American physicians has long been felt; and that the project meets the approbation of the profession and of the relatives of deceased medical men, has already been evinced by valuable contributions from different parts of the country.

The success of the undertaking must obviously depend, in a great measure, upon the co-operation of the medical profession, and such relatives and acquaintances of deceased physicians as may have it in their power to communicate the essential information.

A SPORTING PROJECT.—Several wealthy New York gentlemen have entered into an agreement to purchase a thousand acres of land, and to procure a perpetual lease for another thousand acres, in Pike County, Pennsylvania. There are thousands of acres of wild lands in this county entirely valueless for cultivating purposes, but which abound in wild game of all kinds. It is the object of these gentlemen to own a hunting ground of their own, to stock it thoroughly, and have it protected by law. In a few years they will have the grandest hunting and fishing grounds of America.

—The *Lancet* says: "It is a curious fact that of the passangers in a train which met with a terrible accident lately, all, or very nearly all, who were asleep at the time escaped uninjured—nature's anæsthetic insuring them not only against fractures and contusions, but even against the bad effects of shaking and concussion."

